# 315. Standardized Measurement of SARS-CoV-2 Viral Load Over Time in Respiratory Compartments and in Response to Remdesivir Treatment

**DOI:** 10.1093/ofid/ofac492.393

**Published:** 2022-12-15

**Authors:** Curt Beckwith, Soya Sam, Vladimir Novitsky, Jimin Shin, Kevin Lethbridge, Jon Steingrimsson, Sujata Sahu, Kim Rapoza, Karen Chandran, Vincent J Mariano, Lauri Bazerman, Evelyn R Hipolito, Isabella Diaz, Daniella M Carnevale, Fizza S Gillani, Angela Caliendo, Rami Kantor

**Affiliations:** Alpert Medical School of Brown University, Providence, Rhode Island; Warren Alpert Medical School of Brown University, Providence, Rhode Island; Alpert Medical School of Brown University, Providence, Rhode Island; The Miriam Hospital, Providence, Rhode Island; Rhode Island Hospital, Providence, Rhode Island; Brown University, Providence, Rhode Island; The Miriam Hospital, Providence, Rhode Island; Lifespan, Providence, Rhode Island; The Miriam Hospital, Providence, Rhode Island; Rhode Island Hospital/ The Warren Alpert Medical School of Brown University, Providence, Rhode Island; The Miriam Hospital, Providence, Rhode Island; Rhode Island Hospital, Providence, Rhode Island; Rhode Island Hospital, Providence, Rhode Island; Rhode Island Hospital, Providence, Rhode Island; Brown Alpert Medical School, Providence RI, USA, Providence, Rhode Island; Alpert Medical School of Brown University, Providence, Rhode Island; Alpert Medical School of Brown University, Providence, Rhode Island

## Abstract

**Background:**

Two years into the pandemic, clinicians do not have access to a standardized measurement of SARS-CoV-2 viral load (VL) that allows for VL comparison across clinical specimens and different assays. Reliable VL measurement in diverse respiratory specimens, over time, and in response to treatments such as remdesivir (RDV), could better inform treatment and prevention.

**Methods:**

To investigate the use of a standardized VL assay in respiratory specimens, we enrolled patients hospitalized with COVID-19 in Providence, RI, with/without RDV exposure; collected serial samples from 4 compartments (nasopharyngeal-NP, nasal-NA, oropharyngeal-OP, saliva-SA) in 3 visits during the 1st week of hospitalization; and characterized SARS-CoV-2 VL using a ChromaCode HDPCR™ quantitative research use only assay, calibrated to the first World Health Organization (WHO) International Standard (IS). Linear mixed effects models and associated regression coefficients were used to analyze inter-compartmental VL differences at enrollment, over time, and with/without RDV.

**Results:**

Of 35 participants (60% male; 70% White, 14% Hispanic/Latino, 49% RDV exposure), all had visit 1 samples (median hospital day 1, IQR 0-2; pre-RDV for those exposed); 80% visit 2 samples (median hospital day 2, IQR 1-8); and 37% visit 3 samples (median hospital day 4, IQR 3-7). Overall, 38 NP, 67 NA, 57 OP, and 67 SA samples were collected. Mean log VLs (Log_10_IU/mL) differed by compartment at visit 1 (NP 6.3, NA 4.9, OP 4.1, SA 5.6, p=0.0001) and significantly decreased over time in all compartments (p< 0.04 for all comparisons). Log VL change over time was not significantly different between compartments or between people treated/not treated with RDV.

**Conclusion:**

We successfully measured respiratory intercompartmental SARS-CoV-2 VL differences among hospitalized patients using a standardized assay calibrated to the WHO IS. Dissemination of standardized VL measurement methods will allow accurate VL comparisons across assay types quantified in IU/mL and improve assessment of the impact of COVID-19 treatments. Inter-compartmental VL differences at baseline may indicate sampling variability or different viral burden. RDV did not appear to accelerate viral decay.

**Disclosures:**

**Curt Beckwith, MD**, Gilead Sciences, Inc: Grant/Research Support **Jon Steingrimsson, PhD**, Gilead: Grant/Research Support **Angela Caliendo, MD, PhD**, ChromaCode: Advisor/Consultant|Danaher/Cepheid: Advisor/Consultant|First Light: Advisor/Consultant|Hologic: Grant/Research Support|ID Connect: Advisor/Consultant|Quidel: Advisor/Consultant|Visby: Advisor/Consultant **Rami Kantor, MD**, Gilead Sciences: Grant/Research Support.

